# Requirement of NOX2 Expression in Both Retina and Bone Marrow for Diabetes-Induced Retinal Vascular Injury

**DOI:** 10.1371/journal.pone.0084357

**Published:** 2013-12-17

**Authors:** Modesto Rojas, Wenbo Zhang, Zhimin Xu, Tahira Lemtalsi, Phillip Chandler, Haroldo A. Toque, Robert W. Caldwell, Ruth B. Caldwell

**Affiliations:** 1 VA Medical Center, Augusta, Georgia, United States of America; 2 Vascular Biology Center, Georgia Regents University, Augusta, Georgia, United States of America; 3 Immunotherapy Center, Georgia Reagents University, Augusta, Georgia, United States of America; 4 Department of Pharmacology & Toxicology, Georgia Reagents University, Augusta, Georgia, United States of America; University of Illinois at Chicago, United States of America

## Abstract

**Objective:**

Diabetic retinopathy, a major cause of blindness, is characterized by increased expression of vascular endothelial growth factor (VEGF), leukocyte attachment to the vessel walls and increased vascular permeability. Previous work has shown that reactive oxygen species (ROS) produced by the superoxide generating enzyme NOX2/NADPH oxidase play a crucial role in the vascular pathology. The aim of this work was to identify the cellular sources of the damaging NOX2 activity by studies using bone marrow chimera mice.

**Methods:**

Bone marrow cells were collected from the femurs and tibias of wild type and NOX2 deficient (NOX2^-/-^) donor mice and injected intravenously into lethally irradiated NOX2^-/-^ and wild type recipients. Following recovery from radiation, mice were rendered diabetic by streptozotocin injections. The following groups of bone marrow chimeras were studied: non-diabetic WT→WT, diabetic WT→WT, diabetic WT→NOX2^-/-^, diabetic NOX2^-/-^→WT. After 4 weeks of diabetes, early signs of retinopathy were examined by measuring ROS, expression of VEGF and ICAM-1, leukocyte attachment to the vessel wall and vascular permeability.

**Results:**

The retinas of the diabetic WT→WT chimeras showed significant increases in ROS as compared with the non-diabetic chimeras. These diabetes-induced alterations were correlated with increases in expression of VEGF and ICAM-1, leukocyte adhesion and vascular permeability. Each of these diabetes-induced alterations were significantly attenuated in the diabetic WT→NOX2^-/-^ and NOX2^-/-^→WT chimera groups (p<0.05).

**Conclusion:**

NOX2-generated ROS produced by both bone marrow-derived cells and resident retinal cells contribute importantly to retinal vascular injury in the diabetic retina. Targeting NOX2 in bone marrow and/or retinal cells may represent a novel therapeutic strategy for the treatment/prevention of vascular injury in the diabetic retina.

## Introduction

Diabetic retinopathy, a major cause of blindness in developed countries worldwide [1-3], is characterized by slow and progressive alterations in the retinal microvasculature. The pathology evolves in an environment of increased formation of reactive oxygen species (ROS), leukostasis and breakdown of the blood-retinal barrier which is followed by formation of acellular capillaries and development of micro aneurysms [4-6]. 

Increased ROS formation has been linked to vascular dysfunction and injury in a variety of diseases, including diabetic retinopathy [7-9]. Oxidative stress causes activation of redox-dependent pro-inflammatory transcription factors which can lead to the production of cytokines, chemokines, increased expression of VEGF and up-regulation of adhesion molecules on vascular endothelial cells leading to vascular activation and injury [10]. Early signs of vascular injury during diabetic retinopathy include increased production of VEGF, up regulation of intracellular adhesion molecule-1 ICAM-1[6,11]. VEGF increases ICAM-1 expression on vascular endothelial cells [12] and causes increases in vascular permeability[13]. ICAM-1 mediates the interaction between circulating leukocytes and endothelial cells, leading to increased leukocyte adhesion to the vessel wall [14].

The superoxide generating enzyme NADPH oxidase is a major source of oxidative stress in vascular cells, leukocytes and tissue macrophages. Its activation has been strongly implicated in the vascular complications of diabetes [15-19]. Our previous studies in diabetic mice and high glucose-treated retinal endothelial cells have shown up regulation of ICAM-1 and VEGF, increased leukocyte adhesion to the retinal vessels and increased vascular permeability. All these events were associated with increased expression and activity of the phagocytic NADPH oxidase, NOX2 [6,20]. We also found that deletion of NOX2 or inhibition of NADPH oxidase completely blocked the diabetes-induced increase in ROS production, normalized ICAM-1 and prevented the retinal vascular permeability. Because NOX2 is widely expressed in various types of cells including endothelial cells, smooth muscle cells and leukocytes [21], the specific cellular sources involved in initiating the NOX2-induced vascular injury in the diabetic retina are still unknown.

In this study, we used bone marrow chimeric mice to investigate the tissue sources and role of NOX2 in ROS generation, upregulation of VEGF and ICAM-1 expression and two early indicators of diabetic retinopathy, leukostasis and breakdown of the blood retinal barrier. Our data show that deletion of NOX2 in either bone marrow or retina is effective in limiting the diabetes-induced retinal activation and breakdown of the blood-retinal barrier. This is the first work to show that NOX2 expression in both resident retinal cells and circulating blood cells is required for the initiation of diabetic retinopathy.

## Methods

### Ethics statement

This study was carried out in strict accordance with the recommendations in the Guide for the Care and Use of Laboratory Animals of the National Institutes of Health. The Animal Protocol #2008-245 was approved by the Institutional Animal Care and Use Committee of the Charlie Norwood VA Medical Center. All surgery was performed under anesthesia (Ketamine/Xylazine), and all efforts were made to minimize suffering. 

### Bone marrow chimeras

Male wild-type (WT) and NOX2^-/-^ C57BL/6J mice (Jackson Laboratories, Bar Harbor, ME) were used in these experiments. Groups of 6 week old WT and NOX2^-/-^ mice were irradiated (9.0 Gy) for 10 minutes according to Flaquer and collaborators [22]. Lead shields were used to protect the head during the radiation. On the first day after the radiation, the mice were injected intravenously with fresh bone marrow cells from healthy WT and NOX2^-/-^ mice. Briefly, bone marrow cells were harvested from the femurs and tibias of donor mice, rinsed in essential basal medium (EBM) and a 200 µl cell suspension (1 x 10^7^ nucleated cells) was injected intravenously into each recipient mouse. 

### Mouse model of Type I diabetes

Mice were treated with STZ (75 mg/ kg, i.p., on alternate days for up to 3 injections) beginning at 3 weeks after bone marrow transplantation. Four groups of bone marrow chimeras were studied: non-diabetic WT→WT (n=27), diabetic WT→WT (n=25), diabetic WT→NOX2^-/-^ (n=25), diabetic NOX2^-/-^→WT (n=25). The mice were euthanized at 4 weeks after the onset of diabetes. Average body weight was equal in all groups prior to the induction of diabetes and increased slightly in the control group as compared with the three diabetic groups which did not change during the course of diabetes (Table 1). Diabetes was verified by blood glucose readings above 350 mg/dl (Table 2).

**Table 1:** Body weight (grams, mean+SE,* P<0.05 vs beginning).

Group	WT→WT Control	WT→WT Diabetic	NOX2^-/-^→WT Diabetic	WT→NOX2^-/-^ Diabetic
Beginning weight	21.4 + 0.4	22.6 + 0.5	21.6 + 0.2	22.6 + 0.4
Ending weight	24.0 + 0.6*	21.0 + 0.3	21.2 + 0.2	22.0 + 0.2

**Table 2:** Blood glucose level (mg/dl, mean+SE,* P<0.05 vs control).

Group	WT→WT Control	WT→WT Diabetic	NOX2^-/-^→WT Diabetic	WT→NOX2^-/-^ Diabetic
Blood glucose	180 + 8	552 + 35*	420 + 45*	483 + 15*

### Genotyping

Blood and tissue samples from all groups were collected into tubes containing EDTA and sodium citrate. Genotypes of both blood and tissue samples were tested by using PCR Kit RED Extract-N-Amp ^TM^ Blood PCR Kit from and XNATG Sigma SYBR® Green Extract-N-Amp™ Tissue PCR Kit (Sigma Aldridge). Results for both donor and recipient groups were compared.

### White cell counts

White blood cells were stained with trypan blue (0.4%) and the numbers of white blood cells were counted using a hemacytometer. The investigators were blinded to the experimental groups. 

### Superoxide

To analyze the formation of superoxide in retinal sections the oxidative fluorescent dye dihydroethidium (DHE) was used following the same protocol described in our previous studies [6,20]. 

### Western blot

Western blotting was performed following the method used in our previous study [23]. Retinas were homogenized in a modified RIPA buffer (20 mM Tris-HCl [pH 7.4], 2.5 mM EDTA, 50 mM NaF, 10 mM Na_4_P_2_O_7_,1% Triton X-100, 0.1% sodium dodecyl sulfate, 1% sodium deoxycholate, and 1 mM phenyl methyl sulfonyl fluoride), and 50µg protein samples were separated by 10% sodium dodecyl sulfate polyacrylamide gel electrophoresis, transferred to nitrocellulose membrane, and reacted for 24 hrs with anti-ICAM-1 (rabbit antibody, 1:200, Santa Cruz Biotechnology), anti-VEGF (rabbit antibody, 1:1000, Abcam Laboratories) and anti-albumin (rabbit antibody, 1:5000, Bethyl Laboratories), followed by horseradish peroxidase-linked secondary antibody and enhanced chemiluminescence (GE Healthcare). Equal loading was verified by stripping the membrane and reproving for antibodies against β-actin or tubulin.

### Leukostasis

Leukostasis was assessed by Concanavalin A labeling and quantitation of adherent leukocytes using the method first described by Joussen et al. [14] with slight modifications [6,24]. Briefly, mice were perfused transcrardially with 10 mL of phosphate-buffered saline (PBS) to wash out nonadherent blood cells, followed by perfusion with 10 mL fluorescent isothiocyanate (FITC)–labeled concanavalin A (Con A) lectin (40 μg/mL in PBS, pH 7.4; Vector Laboratories, Burlingame, CA) to label the adherent leukocytes and vascular endothelial cells. Residual unbound Con A was removed by perfusion with PBS. The eyes were removed, fixed with 4% paraformaldehyde and retinal flat mounts were prepared. The flat mounts were imaged using a fluorescence microscope and adherent leukocytes were counted. The Con A positive blood cells were identified as leukocytes based on their size (7 – 20 µm diameter), morphology (round or oval shape) and intraluminal position (as verified by focusing through the vessel). Identity of the Con A positive adherent blood cells as leukocytes was verified by double labeling with the leukocyte marker CD45 [6,24]. 

### Retinal vascular permeability

Vascular permeability was tested by measuring extravasation of albumin into the retinal parenchyma. A thoracotomy was performed on deeply anesthetized mice. The right atrium was cut and a heparinized catheter was inserted into the left ventricle. Mice were perfused transcardially with citrate buffer pH: 4 at 37° C for 6 min to wash out blood cells and proteins. The right eye balls were lysed and Western blotting was performed to quantify retinal vascular leakage by measuring extravascular albumin in the whole retina. 

### Immunolocalization

Frozen retinal sections (10 µm) were fixed with 4% paraformaldehyde for 15 minutes, washed 3 time with PBS and permeabilized and blocked with a solution containing Triton X 100 (0.3% in PBS with normal goat serum 5%). To detect albumin extravasation into the retinal parenchyma, frozen sections were collected from perfused chimeric mice prepared as described above and reacted with a rabbit anti-mouse albumin antibody (1:400, Bethyl Laboratory) followed by reaction with Oregon green labeled goat anti-rabbit antibody (1:500; Invitrogen). To detect diabetes-induced tyrosine nitration in circulating blood cells and retinal tissue, frozen retina samples from non-perfused chimeric mice were reacted with Texas red conjugated isolectin B_4_ at dilution 1:200 for 24 hrs followed by incubation with a rabbit anti-mouse nitrotyrosine antibody from Millipore laboratory at dilution 1:300 for 24 hrs. Another set of samples from non-perfused mice were reacted with isolectin B_4_ as described above and then reacted with blue fluorescent Avidin (1:1000, 1 hr). After washing 3 times with PBS the samples were incubated with rabbit anti-mouse nitrotyrosine antibody as above and CD45 rat anti-mouse antibody (1:300, 24 hrs) and then reacted with Oregon green-labeled goat anti-rabbit and Oregon texas red-labeled goat anti-rat, respectively. Slides were washed 3 times with PBS and covered with anti-fade medium (Vector Laboratories). Images were collected with a fluorescence microscope (Axiovision; Carl Zeiss Meditec, Inc., Dublin, CA)

### Statistical analysis

Group differences were evaluated by using one way analysis of variance of ranks. Results were considered significant at P< 0.05. Data are presented as the mean + SE.

## Results

### Chimeric mice

**Figure 1:**
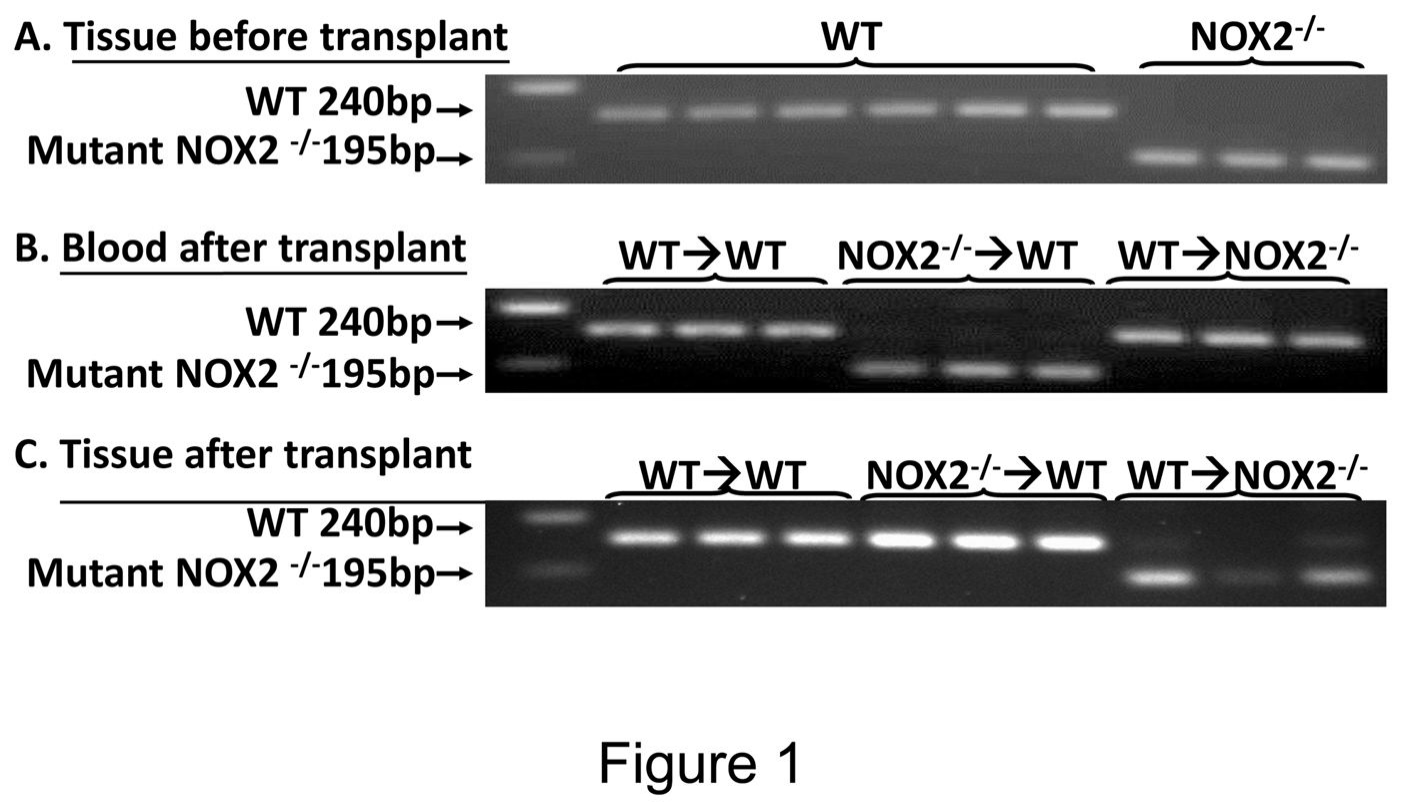
Donor genotype determines the genotype of blood cells in recipient mice. **A**. Genotype of tissue samples from wild type (240 bp) and NOX2^-/-^ mice (195 bp) before transplant. **B**. Genotype of blood samples from recipient wild type (240 bp) and NOX2^-/-^ mice (195 bp) after bone marrow cell transplantation show a band corresponding to the donor genotype. **C**. Genotype of tissue samples from recipient mice after bone marrow transplant show no changes.

PCR analysis was used to examine the NOX2 genotype of tissue and circulating blood cells of the chimera mice. The WT NOX2 has a mobility of 240 bp on DNA gels whereas the mutant NOX2 from NOX2^-/-^ mice migrates with a mobility of 195 bp. This analysis showed that the NOX2 genotype in retinal cells of the chimera mice was not altered by the radiation treatment and bone marrow transplant. However, the NOX2 genotype in the blood cells in each group of recipient mice was converted to that of the donor mice. Thus, the genotype of the blood cells of WT mice that received bone marrow transplants from WT mice remained WT for NOX2 (240 bp). However, blood cells of WT type recipient mice that received bone marrow from the NOX2^-/-^ mice showed the mutant NOX2^-/-^ genotype (195 bp). Similarly, blood cells of the NOX2^-/-^ recipient mice that received bone marrow from WT mice showed the WT NOX2 genotype (240 bp). These results indicate that the NOX2 genotype of blood cells is determined by the genotype of the donor (Figure 1). 

Possible effects of the NOX2 deletion on engraftment and function of the bone marrow cells in the irradiated mice were assessed by white blood cell counts performed at the end of the experiments. The average numbers of white blood cells in each treatment group were as follows: control WT→WT = 5.4 + 0.6 x10^3^/mm^3^, diabetic WT→WT = 6.5 + 0.9 x10^3^/mm^3^, diabetic NOX2^-/-^→WT= 6.4 + 0.8 x10^3^/mm^3^, diabetic WT → NOX2^-/-^ = 6.7 + 0.8 x10^3^/mm^3^. These values are within the normal range reported for mice (5 – 12.0 x 10^3^/mm^3^) [25]. 

### Oxidative stress

**Figure 2:**
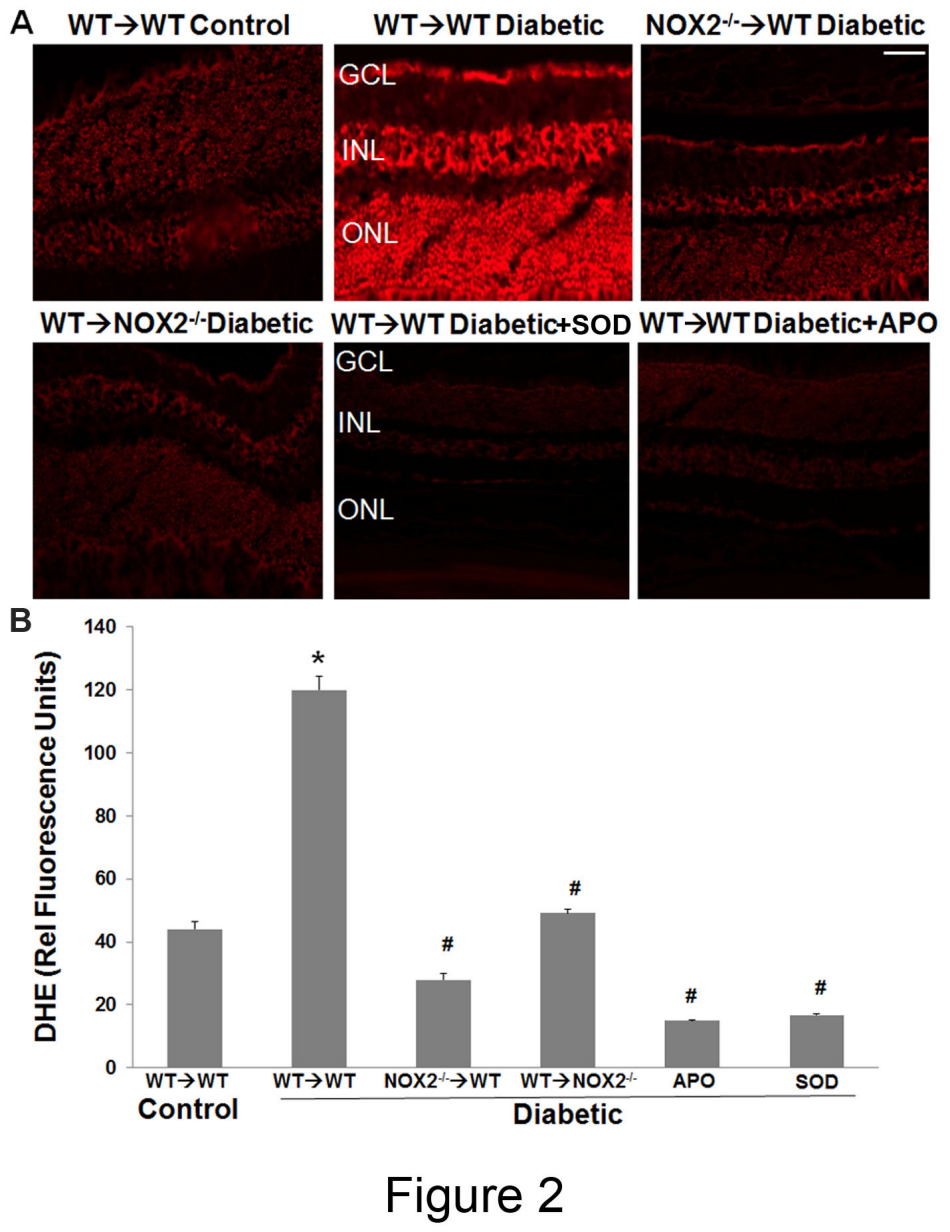
Diabetes-induced increases in ROS are suppressed in NOX2^-/-^ chimeras. **A**. Real time DHE imaging of superoxide formation in retinas from diabetic WT→WT chimeras mice show a strong reaction to DHE as compared with control WT→WT chimeras, the reaction is almost completely abolished in diabetic NOX2^-/-^→WT and WT→NOX2^-/-^ chimeras. Diabetes-induced increase in superoxide production is also abolished by pretreatment with SOD (400U/mL) or apocynin (1 mM). GCL = ganglion cell layer, INL = inner nuclear layer, ONL = outer nuclear layer. **B**. Quantitative analysis of fluorescence intensity in DHE images shows a significant increase in superoxide formation in diabetic WT→WT chimeras. Superoxide production is significantly reduced in diabetic WT→NOX2^-/-^ and NOX2^-/-^→WT chimeras as compared with the diabetic WT→WT chimeras. Pretreatment with apocynin or SOD blocked the DHE reaction. Data represent mean + S.E., *=P<0.001 vs. WT→WT control mice. #=P<0.001 vs WT→WT diabetic mice, n=4, scale bar = 20 μm.

NOX2 is expressed in circulating leukocytes and resident retinal cells and is increased in both sources during diabetes [6,20,26]. We assessed the involvement of bone marrow-derived cells versus retinal cells in superoxide production in the diabetic retina by dihydroethidium (DHE) imaging of frozen retinal sections from the chimera retinas. The sections from diabetic WT→WT chimeras showed a prominent and statistically significant increase in superoxide production as compared with control WT→WT mice (Figure 2A, 2B). This effect of diabetes was blocked in the NOX2^-/-^→WT and WT→NOX2^-/-^ chimeras. Superoxide production in both groups was similar to that in the non-diabetic controls, indicating that NOX2 expression in both bone marrow-derived cells and resident retinal cells is required for diabetes-induced increases in retinal superoxide formation. Control studies showing that addition of apocynin or superoxide dismutase (SOD) prevented the diabetes-induced increase in superoxide formation demonstrated the specificity of the DHE reaction for superoxide and NADPH oxidase activity. 

### Peroxynitrite formation

**Figure 3:**
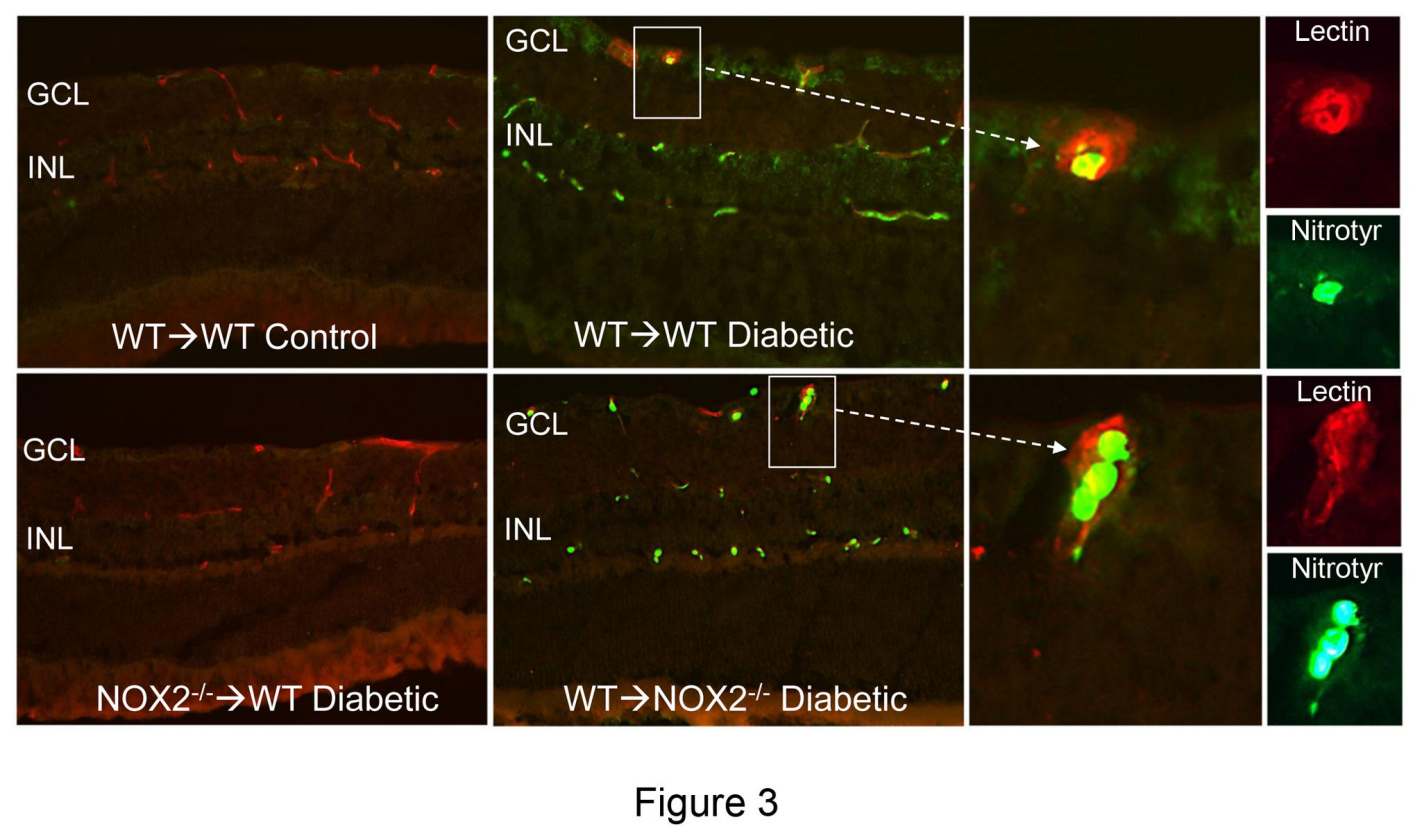
Diabetes-induced increase in tyrosine nitration is blocked in NOX2^-/-^ chimeras. **A**. Double labeling of frozen sections with the vascular marker islolectin B_4_ (red) and anti-nitrotyrosine antibody (green) shows high levels of nitrotyrosine immunoreactivity in the ganglion cell and inner nuclear layers and within and around the retinal vessels (arrows) in the diabetic WT→WT chimeras. Nitrotyrosine immunoreactivity is weak or absent within the retinas of the control WT→WT chimeras and in the retinal parenchyma of both NOX2^-/-^→WT, WT→NOX2^-/-^ diabetic chimeras. However blood cells within the vessels are nitrotyrosine positive in the diabetic WT→NOX2^-/-^ chimeras. Scale bar = 40 μm, GCL = ganglion cell layer, INL = inner nuclear layer. **B**. High power images of the boxed areas in A show nitrotyrosine-positive blood cells (green) within the lectin-labeled blood vessels (red). Scale bar = 25 μm.

**Figure 4:**
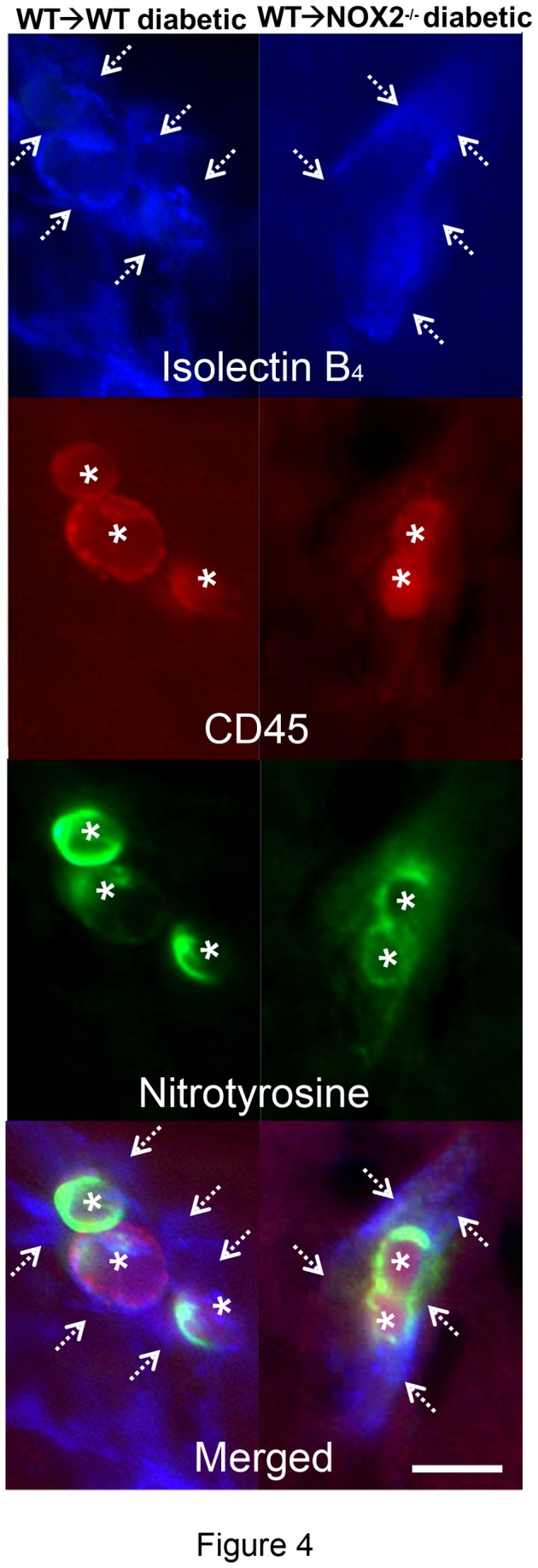
Diabetes-induced tyrosine nitration is localized to CD45-positive leukocytes. Triple labeling for nitrotyrosine (green), CD45 (red) and isolectin B_4_ (blue) shows high levels of nitrotyrosine immunoreactivity on CD45-positive leukocytes (asterisks) localized to the lectin-labeled retinal vessels (arrows), scale bar = 30 µm.

Previous work has shown that diabetes-induced increases in oxidative stress, upregulation of VEGF and ICAM-1 expression, vascular permeability increases and leukostasis are all associated with peroxynitrite formation as shown by increases in immunoreactivity for its biomarker nitrotyrosine [27,28]. Peroxynitrite treatment has also been shown to increase VEGF expression [29]. Thus, we examined the effects of diabetes on peroxynitrite formation by immunolocalization analysis of nitrotyrosine. This analysis confirmed that protein tyrosine nitration was markedly increased in the retinas of the diabetic WT→WT chimeras as compared with the non-diabetic WT→WT controls (Figure 3A). The nitrotyrosine immunoreactivity was localized mainly in the cells located in the inner retina and within vessels of the inner retina and plexiform layers. The nitrotyrosine immunostaining was most intense in the WT→WT diabetic retina and was largely absent in the WT→WT control and NOX2^-/-^→WT diabetic retinas. The lectin-positive blood vessels in the WT→WT and WT→NOX2^-/-^ diabetic chimeras were strongly immunoreactive for nitrotyrosine (Figure 3). The nitrotyrosine-positive blood cells were identified as leukocytes based on their immunoreactivity for the leukocyte marker CD45 (Figure 4). These data suggest that NOX2 expression in bone marrow-derived cells is involved in diabetes-induced increases in peroxynitrite formation within the retinal vessels.

### Leukocyte adhesion and breakdown of the blood-retinal barrier

**Figure 5:**
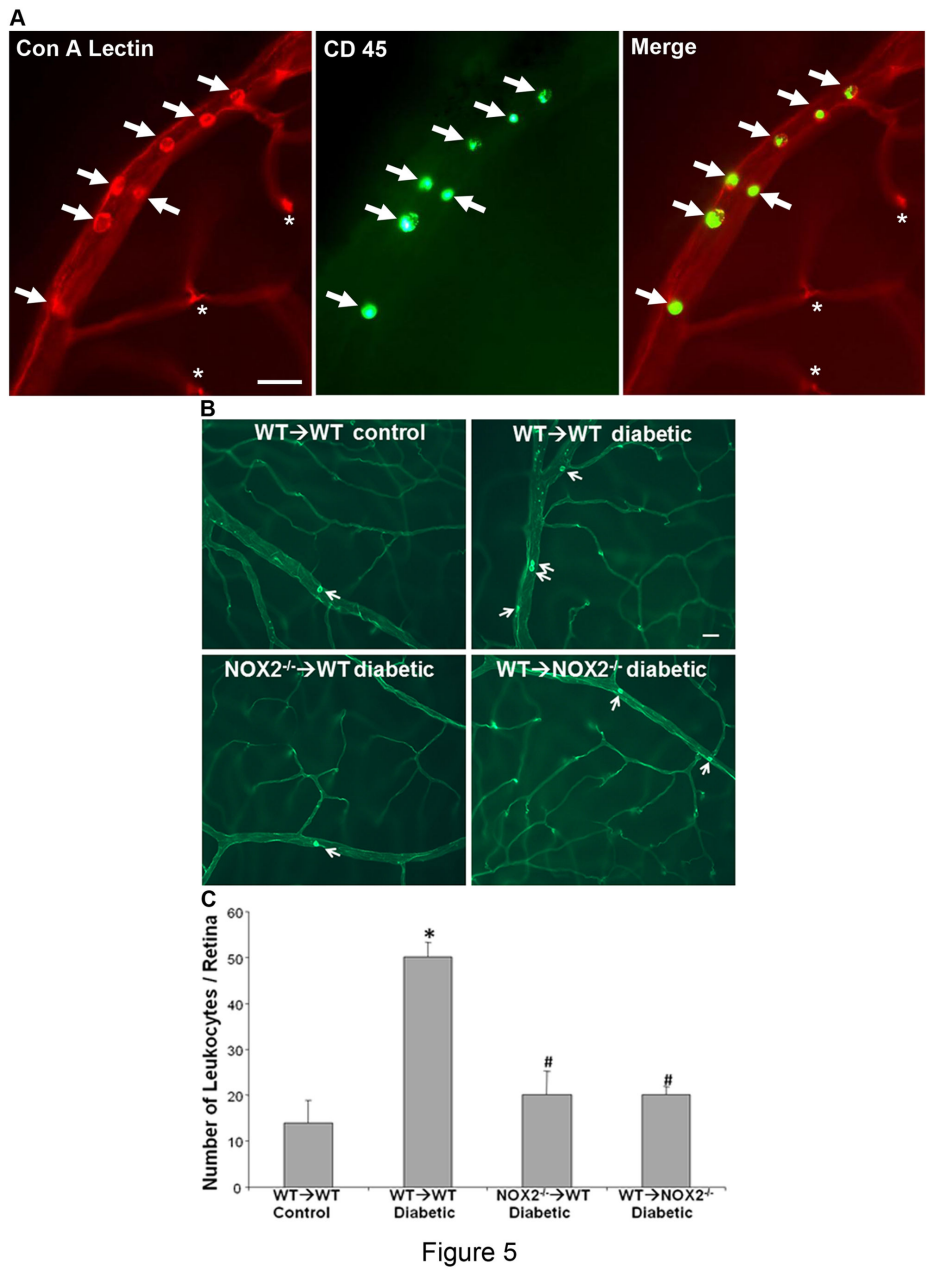
Diabetes-induced increases in leukocyte attachment to the vessel wall are blocked in NOX2^-/-^ chimeras. **A**. Flat mount images showing Con A-labeled vessel containing numerous CD 45-positive 7 - 20 µm diameter leukocytes (arrows). Note that vascular bifurcation areas (asterisks) are also lectin positive, but are small, irregular in shape and negative for CD 45. Thus, leukocytes are easily identified during microscopic imaging based on their size, morphology and location. Scale bar = 50 µm. **B**. Flat-mount images of Concanavalin A-labeled retinas show numerous adherent leukocytes (arrows) within the retinal vessels of diabetic WT→WT chimera. Few leukocytes are present in the control WT→WT or diabetic NOX2^-/-^→WT and WT→NOX2^-/-^ chimeras. Scale bar = 40 μm. **C**. Quantitative analysis shows a significant increase in leukocyte adhesion in the diabetic WT→WT chimeras. This increase is blocked in the NOX2^-/-^→WT and WT→NOX2^-/-^ chimeras. Data represent mean + S.E., *=P<0.001 vs. WT→WT control, ^#^=P<0.05 vs WTWT diabetic, n=12.

Our previous studies have shown that diabetes-induced increases in superoxide are associated with breakdown of the blood-retinal barrier and increased leukocyte attachment to the vessel wall [6]. In the present study we examined the effects of diabetes on leukostasis in chimeric mice by vascular perfusion to remove non-adherent blood cells followed by Con A labeling of leukocytes attached to the vessel wall and microscopic imaging. The identity of the Con A positive blood cells as leukocytes was confirmed by double labeling with anti-CD45 (Figure 5A). The results of this assay showed significant increases in leukocyte attachment to the vessel walls as compared to the non-diabetic WT→WT controls (Figure 5). The diabetes-induced leukocyte attachment was significantly reduced in both WT→NOX2^-/-^ and NOX2^-/-^→WT chimeras. There was no significant difference between the latter two groups. Thus, deletion of NOX2 in either bone marrow derived or retinal cells appears to be sufficient to prevent diabetes-induced leukostasis within the retinal vessels. 

**Figure 6:**
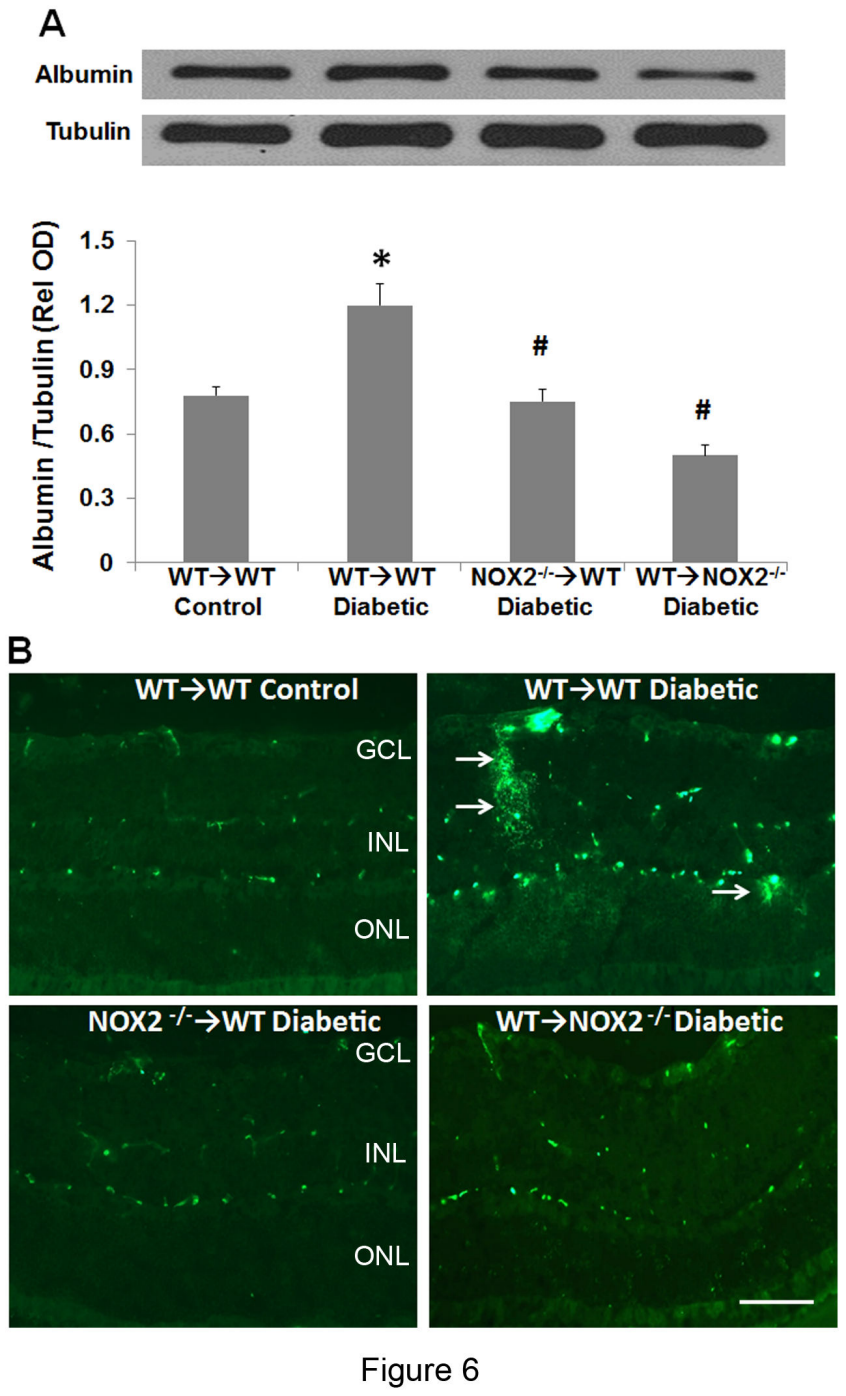
Diabetes-induced breakdown of the blood-retinal barrier is blocked in NOX2^-/-^ chimeras. **A**. Western blot analysis shows a significant increase in extravascular albumin in retina extracts from diabetic WT→WT chimeras as compared with the WTWT control chimeras. This increase is markedly blunted in the NOX2^-/-^→WT and WT→NOX2^-/-^ chimeras. Data represent mean + S.E., *=P<0.05 vs. WT→WT control, ^#^=P<0.05 vs WTWT diabetic, n=6. **B**. Immunolabeling of retinal sections shows prominent leakage of albumin into the retinal parenchyma of the diabetic WT→WT chimera (arrows). The extravasation of albumin is rarely observed in the diabetic NOX2^-/-^→WT and WT→NOX2^-/-^ chimeras, which show a pattern similar to the non-diabetic chimeras. Scale bar = 40 μm, GCL = ganglion cell layer, INL = inner nuclear layer, ONL = outer nuclear layer.

Next we assessed the integrity of the blood-retinal barrier by examining the extravasation of mouse albumin from the retinal vessels. Under normal conditions, where the blood-retinal barrier is intact albumin is confined within the retinal vessels. However under conditions of blood-retinal barrier dysfunction, albumin can leak out into the retinal parenchyma [30]. Western blot analysis showed a significant increase in retinal albumin levels in diabetic WT→WT mice as compared to non-diabetic WT→WT controls. The increase in retinal albumin levels was significantly attenuated in WT→NOX2^-/-^ and NOX2^-/-^→WT chimeras (Figure 6A). These results were comparable and consistent with the results of immunolocalization studies performed in the contralateral eyes of the same mice. The retinas of diabetic WT→WT mice showed prominent increases in extra-vascular albumin as compared with the control non-diabetic WT→WT mice. Extravasation of albumin was markedly attenuated in the diabetic NOX2^-/-^→WT and WT→NOX2^-/-^ chimeras (Figure 6B), suggesting that NOX2 activation in both resident retinal cells and bone marrow derived cells is required for diabetes induced breakdown of the blood-retinal barrier. 

### VEGF and ICAM1 protein expression

**Figure 7:**
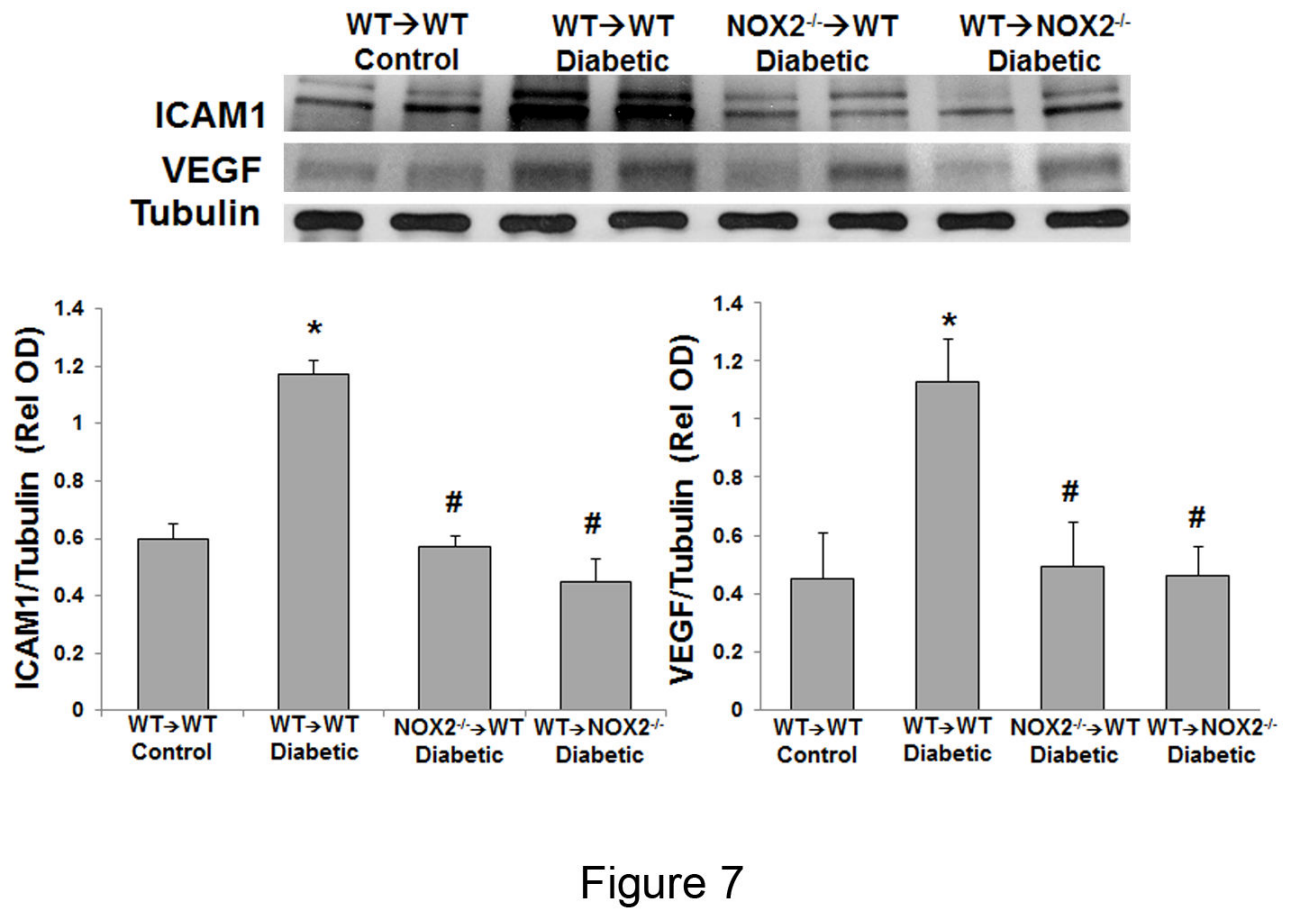
Diabetes-induced increases in ICAM1 and VEGF are blocked in NOX2^-/-^ chimeras. Western blot analysis of retina samples from diabetic WT→WT chimeras shows that ICAM1 and VEGF are significantly increased as compared with the control WT→WT chimeras. This effect is markedly blunted in the diabetic NOX2^-/-^→WT and WT→NOX2^-/-^ chimeras. Data represent mean + S.E., *=P<0.05 vs. WT→WT control, ^#^=P<0.05 vs WT→WT diabetic, n=4.

Diabetes-induced increases in leukostasis and breakdown of the blood-retinal barrier have been shown to be mediated by increases in VEGF and ICAM1 expression. We therefore used western blotting to determine the effects of diabetes on levels VEGF and ICAM1 in the NOX2 chimeras. This analysis showed that both proteins were significantly increased in retinas of diabetic WT→WT chimeras as compared to non-diabetic WT→WT chimeras. The diabetes-induced increases in both VEGF and ICAM1 were markedly attenuated in the NOX2^-/-^→WT and WT→NOX2^-/-^ chimeras (Figure 7). There was no significant difference between the latter two groups in terms of either VEGF or ICAM1 expression. These results indicate that activation of NOX2 in both bone marrow derived cells and retinal cells is required for diabetes-induced increases in expression of VEGF and ICAM1. 

## Discussion

Our results demonstrate that activity of NOX2 NADPH oxidase in both bone marrow-derived blood cells and resident retinal cells contributes to the early signs of retinal vascular injury in diabetic retinopathy by increasing ROS formation. This increase in oxidative stress is associated with increased expression of ICAM1 and VEGF, increased leukocyte adhesion to the vessel wall and breakdown of the blood-retinal barrier. We found that deletion of NOX2 in either bone marrow or retina significantly limits these diabetes-induced alterations. 

During diabetes leukocytes and retinal cells are known to produce high levels of ROS [26,27,31,32]. Leukocytes have been postulated to initiate retinal vascular injury and early signs of diabetic retinopathy in mice [33]. However, we found that diabetes-induced increases in retinal ROS production and signs of diabetic retinopathy were significantly inhibited by deletion of the NOX2 gene in either bone marrow or retina. Thus, expression of NOX2 in both circulating leukocytes cells and retinal tissue is required to increase oxidative stress levels within the retina and induce retinal alterations. This suggests that a reciprocal regulation between retinal cells and leukocytes contributes to the pathogenesis of diabetic retinopathy and activation of the NOX2 NADPH oxidase in both cell types is a required for the propagation of ROS-mediated injury during diabetes.

During diabetic retinopathy, increases in superoxide production have been associated with decreased tissue levels of NO [34,35]. Under normal conditions superoxide levels are controlled by superoxide dismutase (SOD). However superoxide reacts with NO to produce peroxynitrite at a rate three times faster than it can react with SOD. Under oxidative stress conditions, this will lead to a net decrease in NO with an increase in peroxynitrite [36,37]. Peroxynitrite is a major contributor to cellular injury either through direct oxidative reactions with lipids, DNA and proteins or indirect, radical-mediated mechanisms [38]. These reactions trigger cellular responses ranging from subtle modulations of cell signaling to overwhelming oxidative injury, leading to necrosis or apoptosis. Protein tyrosine nitration is now recognized as a biomarker for peroxynitrite formation and has been linked to vascular injury and dysfunction in multiple diseases, including diabetic retinopathy [28,38]. Our immunolocalization studies revealed prominent increases in nitrotyrosine immunoreactivity in the diabetic WT→WT chimera retinas as compared with the controls. The nitrotyrosine immunoreactivity was localized to the retinal vessels, blood cells within the vessels and cells of the retinal ganglion cell layer and inner nuclear layer. The nitrotyrosine signal was weak in the NOX2^-/-^→WT chimeras. By contrast, the diabetic WT→NOX2^-/-^ chimeras displayed a strong reaction in blood cells inside the vessels, but staining in the retinal parenchyma was weak. This suggests that nitrosative cell stress was restricted to circulating blood cells in these mice. 

Pro-inflammatory processes within bone marrow-derived cells play a critical role in the development of diabetic complications [33]. We have shown previously that increases in ROS generated from NOX2 activity are associated with up-regulation of ICAM1 [6]. ICAM1 is constitutively produced by endothelial cells, but its expression is increased by proinflammatory processes [14]. In this study we confirm that diabetes-induced increases in ROS production are associated with an increase in ICAM1 expression. Additionally we show that this process involves NOX2 expression in both bone marrow-derived cells and resident tissue cells (Figure 7). ICAM1 up-regulation is correlated with increased leukocyte adhesion which was completely blocked by deletion of the NOX2 gene in either bone marrow or resident retinal cells. 

A previous study suggested that ICAM1 plays an important role in VEGF-induced increase retinal vascular permeability [13]. Our results indicate diabetes-induced up-regulation of ICAM1 is associated with VEGF up-regulation. VEGF is up-regulated in patients with diabetic retinopathy and is a causative factor of early breakdown of the blood retinal barrier [39,40]. Here, we show that diabetes-induced increase in VEGF expression is associated with significant increases in ROS generation, increases in ICAM1 and abnormal accumulation of albumin outside of retinal vessels. We demonstrated that deletion of the NOX2 gene either in bone marrow or retinal cells is associated with decreases in ROS formation, prevention of increases in ICAM1 and VEGF increases, decreased leukostasis and preservation to the blood-retinal barrier. 

To our knowledge this study is the first to show that expression of NOX2 in both bone marrow-derived cells and resident retinal cells is required to initiate ROS production and activate the downstream inflammatory events associated with the early signs of diabetic retinopathy. Although we did not identify specific cell sources of ROS production, our data clearly show that both circulating bone marrow-derived cells and resident retinal cells are involved. A potential limitation of studies using diabetic chimeras is that diabetes has been suggested to affect hematopoiesis [41]. Although our analyses showed equal numbers of white blood cells in all groups, we cannot rule out potential effects of diabetes on the distribution of white blood cell populations. However, our finding that both WT→NOX2^-/-^ and NOX2^-/-^→WT diabetic chimeras showed similar decreases in ROS production and signs of retinopathy as compared with the diabetic WT→WT chimeras and that the values in both diabetic mixed chimeras resembled those in the non-diabetic controls suggests that the bone marrow cells engraftment was successful. Moreover, a previous study showed a protective effect of iNOS or PARP1-deficient bone marrow cells on retinal inflammation and acellular capillary formation in mice rendered diabetic two weeks prior to the bone marrow transplant [33]. 

In summary our results provide the first evidence of the requirement for NOX2/NADPH oxidase activity in both bone marrow-derived cells and resident retinal cells for generating ROS, leading to increases in VEGF and ICAM1 expression, increased leukocyte adhesion and breakdown of the blood retinal barrier. Targeting NOX2 activity in bone marrow and/or retinal resident cells may represent a novel therapeutic strategy for the treatment/prevention of diabetic retinopathy.
